# Large Magneto-piezoelectric Effect in EuMnBi_2_ Single Crystal at Low Temperatures

**DOI:** 10.1038/s41598-020-64530-2

**Published:** 2020-05-05

**Authors:** Yuki Shiomi, Hidetoshi Masuda, Hidefumi Takahashi, Shintaro lshiwata

**Affiliations:** 1 Department of Basic Science, University of Tokyo, Meguro Tokyo, 153-8902 Japan; 2 Department of Applied Physics, University of Tokyo, Hongo Tokyo, 113-8656 Japan; 3 Institute for Materials Research, Tohoku University, Sendai, 980-8577 Japan; 4 Division of Materials Physics, Graduate School of Engineering Science, Osaka University, Osaka, 560-8531 Japan

**Keywords:** Condensed-matter physics, Ferroelectrics and multiferroics, Condensed-matter physics

## Abstract

Magneto-piezoelectric effect (MPE) refers to generation of strain in response to electric currents in magnetic metals which lack both time-reversal and space-inversion symmetries. A recent experimental paper demonstrated the MPE in the antiferromagnetic metal EuMnBi_2_ at 77 K, but the limited temperature range of the MPE measurement hampered detailed discussion on the MPE. Here we extend the measurement temperature range down to liquid He temperature, and studied the dependences of the MPE on the laser position, frequency and amplitude of electric currents, and temperature in the very low temperature range. We show that the MPE signal is enhanced at low temperatures and reaches a maximum magnitude in the antiferromagnetically ordered states of both Eu and Mn ions. An effective piezoelectric coefficient for the MPE at 4.5 K is estimated to be as large as 3500 pC/N, which is much larger than piezoelectric coefficients of typical piezoelectric ceramics, although the magnitude of real MPE displacements should be limited due to strong Joule heating at high electric currents. The present results may open up a new strategy to realize new lead-free piezoelectric materials.

## Introduction

Piezoelectric materials which allow conversion between mechanical stress (strain) and electrical voltage are technologically important^1–4^. The most widely used piezoelectric material in the industry is the lead zirconate titanate (PZT). The piezoelectric coefficient of PZT is known to be as large as ~1000 pC/N at room temperature^5^. Despite its outstanding piezoelectric property, PZT currently faces global restrictions due to Pb toxicity; thus, there is an urgent need to develop a lead-free alternative that can compete with PZT^3,4^. However, its achievement is still challenging, although much effort has been devoted to the development of lead-free piezoelectric materials. A new guideline for searching for efficient piezoelectric materials is awaited^6^.

The magneto-piezoelectric effect (MPE), which was very recently explored theoretically^7,8^ and experimentally^9,10^, provides a new mechanism of piezoelectric responses in low-symmetry magnetic metals. While the usual piezoelectric effect is allowed in noncentrosymmetric insulators and semiconductors, the MPE occurs in noncentrosymmetric metals possessing many conduction electrons. When expressed in mathematical formulas, the (inverse) piezoelectric effect is described by

1 $${\varepsilon }_{ij}={d}_{ijk}{E}_{k},$$

while the MPE may read

2 $${\varepsilon }_{ij}={e}_{ijk}{J}_{k},$$

where *ε*_*ij*_, *d*_*ijk*_, *E*_*k*_, *e*_*ijk*_, and *J*_*k*_ are a strain tensor, piezoelectric coefficient, electric field, MPE coefficient, and electric current, respectively. Since Eq. (1) and Eq. (2) have similar forms in terms of symmetry, constraints on the space inversion symmetry for the MPE coefficient are similar to those for the piezoelectric effect. The above relation means that not the electric field but the electric current is an intrinsic quantity in the MPE. Since *J*_*k*_ is proportional to *E*_*k*_ owing to Ohm’s law, however, the MPE can be regarded as a piezoelectric response^8^. Importantly, a theory^8^ predicts that the MPE is irrelevant to screening which should significantly suppress the piezoelectric responses in metals. Thus, the study of the MPE is a promising research direction toward the realization of new efficient piezoelectric materials. Moreover, coexistence of piezoelectricity and high conductivity is difficult to achieve with conventional piezoelectric materials and the MPE could give rise to new functions in piezoelectric devices.

Up to now, two theoretical mechanisms have been proposed for the MPE: the first mechanism is a Berry-curvature (topological) effect^7^, while the second one was discussed in terms of electronic nematicity^8^. These two mechanisms look different, since an external magnetic field is assumed in the first mechanism, while the second mechanism is validated without magnetic fields. In the second mechanism of the MPE^8^, the authors consider antiferromagnetic metals in which time-reversal and space-inversion symmetries are simultaneously broken. In such low-symmetry magnetic metals, applied electric currents are expected to induce an electronic nematic order^8^; the nematic order accompanies the distortion of the Fermi surface, which then leads to the deformation of lattices through electron-lattice couplings. This mechanism of the current-induced distortion is free from magnetic fields and thus particularly important from the viewpoint of practical applications.

**Figure 1:**
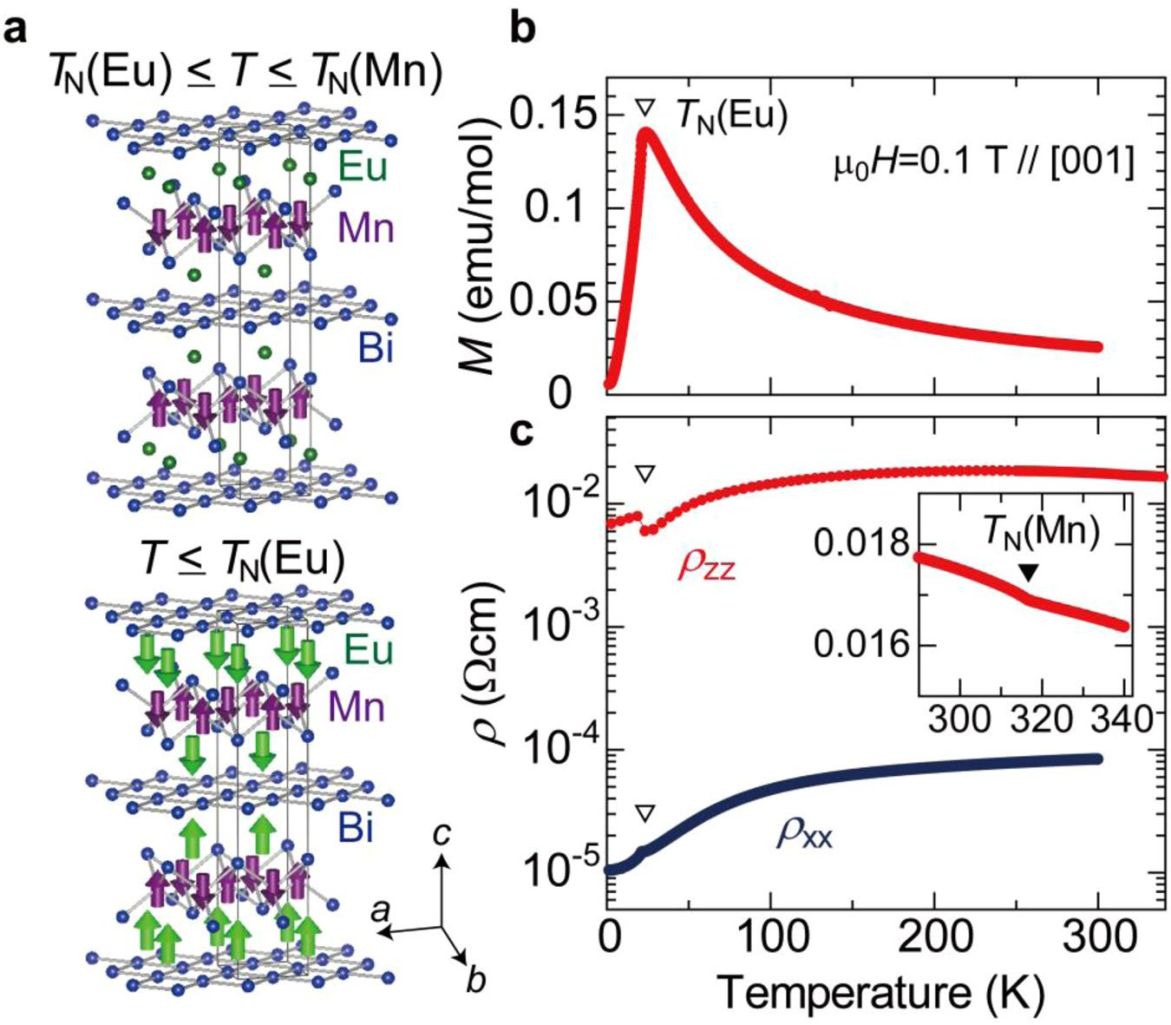
(**a)** Crystal structure of EuMnBi_2_ and its magnetic structure in the temperature (*T*) range of *T*_N_(Eu) ≤ *T* ≤ *T*_N_(Mn) (top row) and *T* ≤ *T*_N_(Eu) (bottom row). Mn magnetic moments are indicated by purple arrows, while Eu moments by green arrows. (**b)** Temperature dependence of the magnetization (*M*). The magnetization was measured in magnetic field of 0.1 T applied along the *c* axis. The open triangle indicates the antiferromagnetic transition temperature of Eu ions, *T*_N_(Eu). (**c**) Temperature dependence of inplane and interlayer resistivities, *ρ*_*xx*_ and *ρ*_*zz*_, respectively. The inset shows the magnified view of the temperature dependence of *ρ*_*zz*_ in the high temperature regime. The open triangles indicate the antiferromagnetic transition temperature of Eu ions (*T*_N_(Eu)) and the closed triangle the antiferromagnetic transition temperature of Mn ions (*T*_N_(Mn)).

Experimentally, the authors of the present work demonstrated the MPE of the second mechanism in the antiferromagnetic metal EuMnBi_2_ at 77 K^9^. Application of AC electric voltage along the [001] direction at 77 K induces an AC displacement signal at the same frequency along the [100] direction, but not along the [001] direction^9^. This direction dependence is consistent with the symmetrical constraint for EuMnBi_2_^8,11^; from the *D*_2d_ symmetry, electric current applied along the [001] direction is expected to generate strain along the [110] direction. Temperature dependent measurements^9^ showed that the MPE signal decreases with increasing temperature and disappears at around 200 K, above which transport along the [001] direction changes from coherent to incoherent. In the incoherent transport regime, the interlayer transport is no longer dictated by the Fermi-liquid picture, and the MPE is indiscernible. The emergence of the MPE signal above 77 K agrees with the symmetry breaking by Mn antiferromagntic structure, as illustrated in the top row of Fig. 1a^12,13^. However, there are two magnetic ions (Mn and Eu ions) in EuMnBi_2_, and in fact, EuMnBi_2_ has the other magnetic transition corresponding to antiferromagnetic ordering of Eu moments at a much lower temperature than 77 K (bottom row of Fig. 1a)^12–14^. Because of the limited temperature range of the MPE measurement conducted with a liquid-nitrogen optistat^9^, influence of antiferromagnetic Eu moments on the MPE remains elusive.

Here, we extend the measurement temperature range down to liquid-He temperature to study in detail the MPE of EuMnBi_2_. Compared to the previous paper^9^, we have improved the following points in the MPE measurement: (i) the lowest temperature is 4.5 K in the present paper, while it was 77 K in the previous paper^9^; (ii) the measurement direction of the MPE displacement signals is set at [110] direction consistent with the theory^8^ in the present experiment, while it was [100] direction^9^ different from the theoretical proposal^8^; (iii) to apply AC electric currents to the sample, we here use a constant current source to precisely estimate the current amplitude flowing inside the sample, instead of a constant voltage source used in the previous paper^9^.

## Results

### Magnetization and resistivity

EuMnBi_2_ is structurally related to 122-type iron pnictide superconductors^14^. The space group is I4/mmm (139)^12,14^ and centrosymmetric. The crystal structure is shown in Fig. 1a together with magnetic structures of Mn and Eu moments^12,13^. EuMnBi_2_ is built from three layers composed of edge-sharing MnBi_4_, Eu, and Bi nets. The layers are stacked along the [001] direction (*c* direction), and electric conduction is known to be highly anisotropic because of the quasi two-dimensional structure in the (001) plane (*ab* plane)^12^. The antiferromagnetic structure of Mn ions (Fig. 1a) break the space inversion symmetry, as discussed for (Ba,K)Mn_2_As_2_ possessing the same crystal symmetry^8^.

Figure 1b shows the temperature dependence of magnetization, *M*, for our single crystal. Here, *M* was measured in the magnetic field of 0.1 T applied along the [001] direction. As temperature decreases from 300 K, the magnetization increases and exhibits a peak at 23 K. This peak temperature corresponds to the antiferromagnetic transition of divalent Eu ions. Above the magnetic transition temperature of Eu ions (*T*_N_(Eu)), Curie-type paramagnetization of Eu ions is observed, and the relatively-small antiferromagnetic moment of Mn ions is not recognized. Below *T*_N_(Eu), the magnetization sharply drops, consistent with the antiferromagnetic order of Eu ions. The overall temperature dependence of the magnetization agrees with previous papers^12,14^.

Temperature dependence of resistivity (*ρ*) is shown in Fig. 1c. Since EuMnBi_2_ possesses the two-dimensional crystal structure, the resistivity is highly anisotropic, as mentioned before. The in-plane resistivity *ρ*_*xx*_ shows usual metallic behavior as shown in Fig. 1c. At 300 K, *ρ*_*xx*_ is approximately 1 × 10^−4^ Ωcm and decreases monotonically with decreasing temperature. The magnitude of *ρ*_*xx*_ at the lowest temperature is approximately 1 × 10^−5^ Ωcm. At *T*_N_(Eu), *ρ*_*xx*_ shows an anomaly as a small peak.

In contrast, temperature dependence of the resistivity measured along the [001] axis (*ρ*_*zz*_) is rather complicated. As temperature decreases from 340 K, *ρ*_*zz*_ first increases, shows a broad maximum at around 200 K, and then decreases below 200 K. The broad peak of the interlayer resistivity indicates incoherent-coherent crossover of electric conduction^15–20^ at ~200 K. Above ~200 K, transport mean free path along the [001] direction is shorter than the interlayer distance, and the hopping conduction becomes dominant. A similar incoherent-coherent crossover was reported in isostructural SrMnBi_2_^21^.

At *T*_N_(Eu), *ρ*_*zz*_ shows a small jump; the anomaly at *T*_N_(Eu) looks larger in *ρ*_*zz*_ than *ρ*_*xx*_. Also, the temperature dependence of *ρ*_*zz*_ shows a small anomaly at 314 K, as shown in the inset to Fig. 1c; this is the antiferromagnetic transition temperature of Mn ions, *T*_N_(Mn). Below *T*_N_(Mn), the antiferromagnetic structure of Mn ions [Fig. 1a] breaks the space inversion symmetry, and the MPE is allowed, as demonstrated at 77 K in our previous paper^9^. We here stress that influence of the antiferromagnetic Eu moments was not studied in our previous paper^9^, since the antiferromagnetic transition temperature of Eu ions is much lower than 77 K. Though the antiferromagnetic structure of Eu ions does not globally break the space inversion symmetry as shown in the bottom row of Fig. 1a, couplings of Eu magnetic moments with Bi conduction electrons and Mn moments are expected to affect the MPE at very low temperatures.

### Laser position dependence of MPE signals at 4.5 K

**Figure 2:**
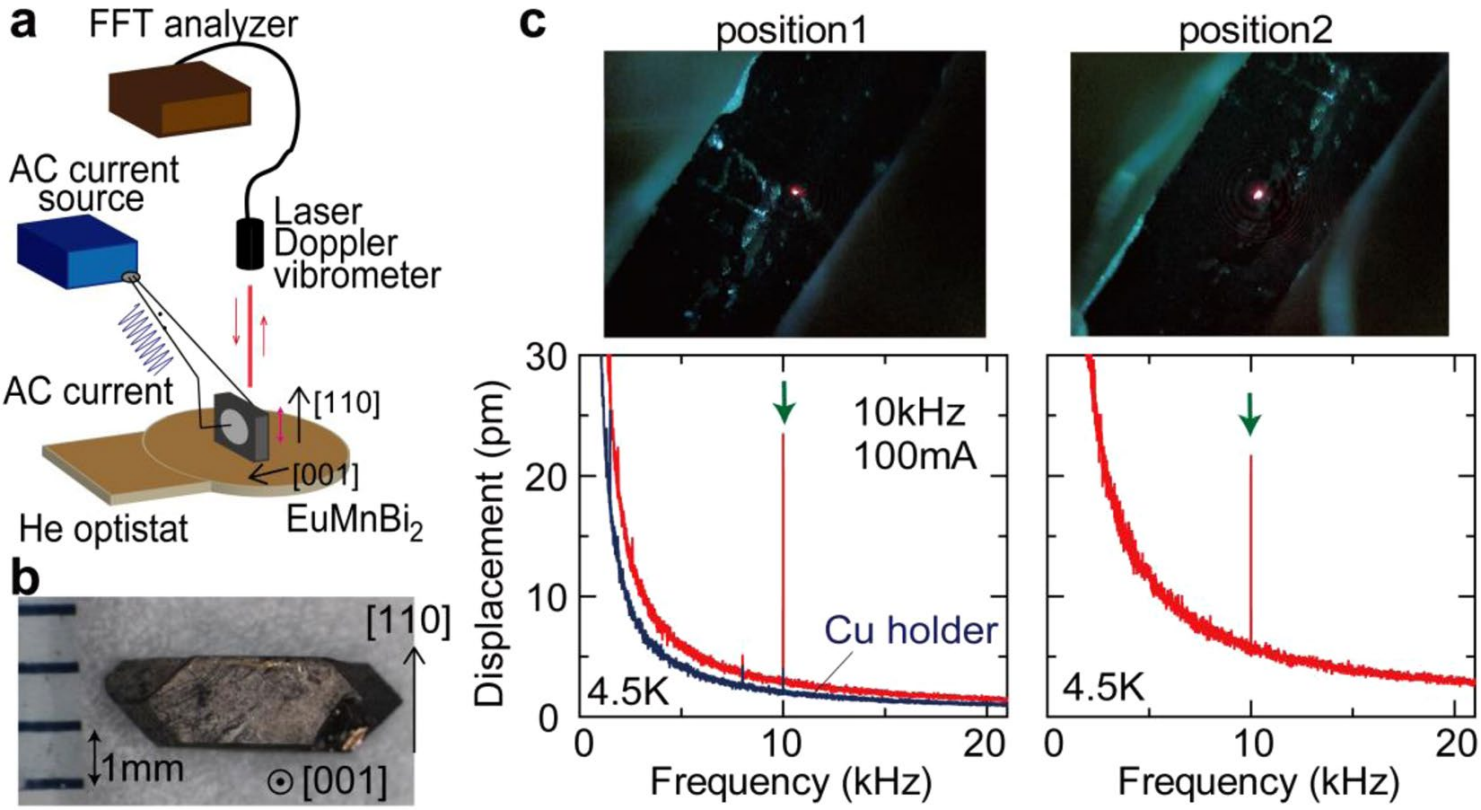
(**a**) Schematic illustration of measurement set-up of the magneto-piezoelectric effect (MPE). A EuMnBi_2_ sample was fixed on a Cu holder of a liquid-He optistat. The lowest temperature of the MPE measurement is 4.5 K. The optistat has a quartz window and the laser is irradiated from a laser Doppler vibrometer to the sample through the quartz window. An AC electric current is applied to the sample along the [001] direction and the AC displacement is measured along the [110] direction. Using the Doppler effect of the laser reflected from the sample, the velocity of sample vibration was extracted. The vibration velocity was transferred to the FFT analyzer and numerically integrated with respect to time to obtain the displacement signal. (**b**) A photograph of the EuMnBi_2_ sample. The largest plane corresponds to the (001) plane. (110) planes are prepared to measure AC displacements along the [110] direction. (**c**) Sample photographs (top row) and frequency spectra of displacement signals (bottom row) at 4.5 K. The MPE measurement was performed at two positions: position-1 and position-2. The red dots in the photographs correspond to the laser spot of the laser Doppler vibrometer. In response to the AC electric current with 10-kHz frequency and 100-mA (zero-to-peak) amplitude, AC displacement signals appear at the same frequency as the AC electric current. The current-induced signals are highlighted by green arrows in the figures. The displacement signals measured on the Cu sample holder are also shown.

We performed the MPE measurement at low temperatures. The measurement system is illustrated in Fig. 2a. We applied AC electric current along the [001] direction, and measured AC displacement signals along the [110] direction. A sample photograph is shown in Fig. 2b. As-grown crystals grow into flat plates with the (001) plane as the largest plane. We cut a sample so as to obtain (110) planes. The sample was fixed on a Cu holder of the liquid-He optistat using GE varnish.

In Fig. 2c, we first measured AC displacements showing up in response to AC electric currents at 4.5 K. To get reliable displacement signals with a laser Doppler vibrometer, the intensity of the reflected light must be sufficiently strong and stable; the displacement signal may depend on conditions of the sample surface (scratch, pinhole, etc.). We then performed the MPE measurement at two different positions of the EuMnBi_2_ sample: position-1 and position-2 in Fig. 2c. In the photographs of Fig. 2c, red dots correspond to the laser spot of the laser Doppler vibrometer. The diameter of the laser spot is several tens of micrometers, which can be smaller than the size of antiferromagnetic domains^22–24^.

Frequency spectra of AC displacements at position-1 and position-2 are shown in the bottom row of Fig. 2c. We here applied the AC electric current with 10-kHz frequency and 100-mA (zero-to-peak) amplitude. In response to the AC electric current, we found that an AC displacement signal appears at the same frequency as the AC electric current (10 kHz). The magnitude of the current-induced displacement is 23.4 pm at position-1 and 21.6 pm at position-2; displacement signals with the similar magnitudes are observed at the different two positions. This signal cannot be attributed to extrinsic vibration of the Cu sample holder. In Fig. 2c, displacement signals measured on the Cu sample holder are also shown (data in blue color). In response to the AC electric current, a tiny signal with the 5-pm magnitude is observed. This tiny signal may originate from electromechanical effects in cables and electrode terminals. The observed displacement signal driven by the AC electric current on the EuMnBi_2_ sample, which is much larger than the artifact signal on the Cu holder, can be safely ascribed to the MPE.

It is noted that background displacements observed at other frequencies than 10 kHz increase with decreasing frequency and diverges toward the zero frequency in Fig. 2c. The divergent frequency dependence in the very low frequency range is attributed to an artifact in the numerical integration in the FFT analyzer. The numerical integration of velocity data with respect to time corresponds to division of velocity by frequency in the frequency domain, and thus the calculated displacement diverges almost in proportion to *1/f (f*: frequency) at low frequencies. Similar frequency dependence of background signals in the MPE measurements was observed at 77 K in previous papers^9,10^. In the following sections, background levels at the electric-current frequency are estimated by fitting the spectra with *A/f + B* (*A* and *B*: fitting parameters).

### Current-frequency and Current-amplitude dependence of MPE signals at 4.5 K

**Figure 3:**
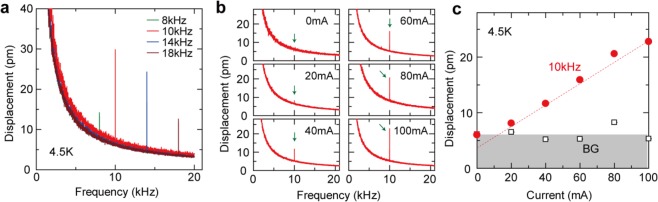
(**a**) Frequency spectra of displacement signals measured at different frequencies of AC electric currents. The measurement temperature is 4.5 K. (**b**) Frequency spectra of displacements at different amplitudes of AC electric currents. The frequency of the AC electric current was kept at 10 kHz. The measurement temperature is 4.5 K. The current-induced signals are highlighted by green arrows. **c**. Current-amplitude dependence of the current-induced displacement signals (red dots) and the background (BG) signals (open squares). The background levels are estimated by the 10-kHz values of the fitted curves for the spectra: *A/f + B*, where *f* is the frequency and *A* and *B* are fitting parameters. The dotted line is just guides for eyes. This plot is obtained from the data in (**b**). The background levels are shaded in gray just for clarity.

We further investigate properties of the current-induced displacement signals at 4.5 K. In Fig. 3a, we changed the frequency of the AC electric current to study the current-frequency dependence of the displacement signals, while the amplitude of the AC electric current was here kept at 100 mA. We found that the current-frequency dependence of the MPE signals is not simple; in Fig. 3a, the magnitude is the largest at 10 kHz, and it decreases at both lower and higher frequencies. At low frequencies, the signal decreases in magnitude because of larger background signals at lower frequencies, while it decreases also at high frequencies since a bonding material (GE varnish) between the sample and the Cu holder affects the sample deformation in a frequency-dependent manner^9,10^. A similar current-frequency dependence as observed in Fig. 3a was reported in the previous paper at 77 K^9^.

We then kept the frequency of the AC electric current at 10 kHz, and changed its amplitude from 0 mA to 100 mA. In Fig. 3b, we show frequency spectra of displacements measured at different amplitudes of AC electric currents. At 0 mA, only background signals are observed, and no displacement signals caused by the electric current are recognized. The small current-induced signal is visible at 20 mA. As the current amplitude increases from 20 mA, the magnitude of the current-induced signal increases monotonically.

The current-amplitude dependence of the displacement signals is summarized in Fig. 3c. Background (BG) levels are estimated by the 10-kHz values of the fitting curves for the spectra (see the previous section), and found to be approximately 5 pm in the entire current range, as displayed by gray color in Fig. 3c. The magnitude of the current-induced signal increases with increasing current amplitudes, and importantly the increase rate is proportional to the amplitude of the electric current, as shown by the dotted line in Fig. 3c. This linear current-amplitude dependence is consistent with the MPE; see Eq. (2). It is noted that we found a small offset (~3 pm) at 0 mA in the linear fit to the experimental data (see the dotted line in Fig. 3c). This offset independent of electric currents should be due to experimental noises^9^. Since the MPE displacement signal is very small, temporal fluctuation of background levels is non-negligible.

### Temperature dependence of MPE signals

**Figure 4:**
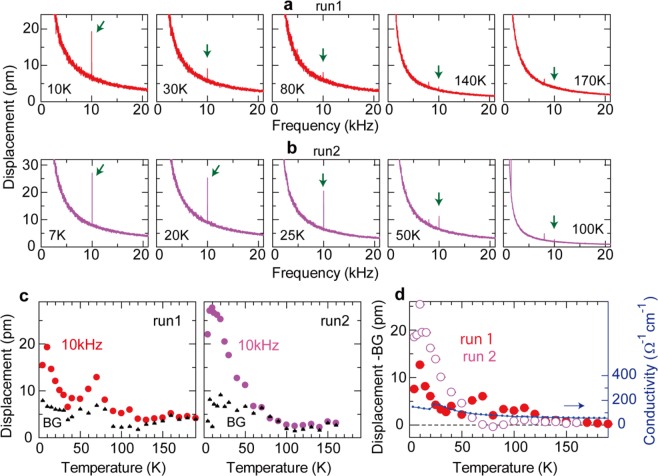
(**a,b**) Frequency spectra of displacements at various temperatures in run-1 (**a**) and run-2 (**b**). The current-induced signals are highlighted by green arrows. (**c**) Temperature dependence of the MPE signals (dots) and background (BG) signals (closed triangles) in run-1 and run-2. These plots are obtained from the data in (**a**,**b**). Background levels are estimated by the 10-kHz values of the fitted curves for the spectra: *A/f + B*, where *f* is the frequency and *A* and *B* are fitting parameters. (**d**) Temperature dependence of the displacement signal at 10 kHz minus the background (BG) signal in run-1 and run-2. The data are obtained from (**c**). Temperature dependence of electrical conductivity measured along the interlayer direction (= 1/*ρ*_*zz*_) is also shown.

Temperature dependences of the MPE signals are studied in two measurement runs: run-1 and run-2 in Fig. 4. The two measurement runs were performed at different laser positions on the sample. Here, we kept the frequency and the amplitude of the AC electric current at 10 kHz and 100 mA, respectively. As mentioned in our previous papers^9,10^, stable measurement of the temperature dependence of small MPE signals is difficult in our experimental set-up, since the laser position and focus inevitably change at different temperatures owing to thermal expansion of the sample and the holder at high temperatures. Nevertheless, the temperature dependence data should be important to clarify the physical mechanism of the MPE, and thus it is discussed in detail by conducting two measurement runs.

In Fig. 4a and Fig. 4b, we plot the frequency spectra of displacements at selected temperatures in run-1 (Fig. 4a) and run-2 (Fig. 4b). In both run-1 and run-2, we found that the MPE signals decrease with increasing temperature and disappear at high temperatures. The signal becomes almost invisible at 140 K in run-1, while it becomes lower than the measurement limit already at 100 K in run-2; the decrease in the MPE signal is more significant in run-2 than run-1. The different temperature dependence between run-1 and run-2 could be attributed to different laser positions; the signal magnitude depends on surface conditions (Fig. 2c) and also it should be different at different magnetic domain structures. Nevertheless, we here stress that the MPE signal in both runs disappears well below the antiferromagnetic transition temperature of Mn ions (314 K). This result is consistent with the previous report where the MPE signal is observed only in the coherent transport regime below ~200 K^9^.

The temperature dependence of the MPE signals at 10 kHz and background signals is summarized in Fig. 4c. Here, background (BG) levels are estimated by the 10-kHz values of the fitting curves for the spectra. The magnitudes of the background levels are scattered at each temperature, but smaller than 10 pm in the entire temperature regime. The MPE signal at 4.5 K has a similar magnitude (~20 pm) in run-1 and run-2, and tends to decrease with increasing temperature although the temperature dependence is not monotonic in run-1. Interestingly, in run-2, the temperature dependence of the MPE signal exhibits a kink at around 20 K, close to the antiferromagnetic transition temperature of Eu ions [*T*_N_(Eu)]. In the low temperature regime where both Eu and Mn moments are antiferromagnetically ordered, the MPE signals seem to have larger magnitudes than in the high temperature regime where only Mn moments are ordered. It is noted that the magnitude of the MPE signal suddenly changes by increasing temperature from 4.5 K; in run-2, we confirmed that the MPE signal jumps when the temperature is changed from 4.5 K to 7 K. In our measurement system, temperature fluctuation is smaller than 0.1 K at the lowest temperature of 4.5 K, while it is <1 K at different temperatures where the temperature is controlled using a heater attached beneath the Cu sample holder. The temperature stability might be related to the observed magnitude of the MPE signal e.g. through fluctuation of laser positions.

It was theoretically pointed out that the magnitude of the MPE signal is proportional to scattering time. To check this relationship, we plot the temperature dependence of the magnitude of the MPE signal along with that of the interlayer conductivity (= 1/*ρ*_*zz*_ ∝ scattering time) in Fig. 4d. Here, the background signals are subtracted from the MPE signals to rule out the temperature dependence of the background signals. We found that the temperature dependence of the MPE signal almost follows that of the interlayer conductivity in the high temperature regime, but below 50 K, the MPE signal rapidly grows unlike the case for the interlayer conductivity. This result suggests that Eu antiferromagnetic moments play a role on the enhancement of the MPE signal. In terms of the symmetry consideration, Eu magnetic moments have no direct contribution to the MPE, since the magnetic structure of Eu ions does not break the space inversion symmetry (Fig. 1a). However, the MPE depends on many other physical parameters, e.g. electron-lattice coupling and spin-exchange coupling between itinerant Bi-electron spins and localized Mn moments^9^. The spin-exchange coupling around and below *T*_N_(Eu) can be modified by a hybridization of Eu moments with Mn moments and conduction electron spins, presumably giving rise to the enhancement of the MPE signal.

## Discussion

The efficiency of the MPE can be measured by an effective piezoelectric coefficient: $\tilde{d}$ = *ε*[110]/*E*_*z*_ = *ε*[110]/(*ρ*_*zz*_ *j*), where *ε*[110], *E*_*z*_, *ρ*_*zz*_, and *j* are strain, electric field, interlayer resistivity, and electric current density, respectively. When we use 20 pm as a representative magnitude of the MPE signal at 4.5 K, we obtain *ε*[110] = 20 pm/1.4 mm = 1.4 × 10^–8^. The electric field inside the EuMnBi_2_ sample is estimated to be *E*_*z*_ = 10^−2^ Ωcm × 100 mA/2.5 mm^2^ = 4.0 V/m. By dividing *ε*[110] by *E*_*z*_, we obtain $\tilde{d}$ = 3500 pC/N at 4.5 K.

The estimated $\tilde{d}$ value is extremely large compared to room-temperature piezoelectric coefficients of conventional piezoelectric ceramics (*d* ≲ 1000 pC/N)^5^, although real MPE displacement signals should be limited to small magnitudes because of limited magnitudes of electric fields inside metallic samples. If very high electric current is applied to metallic samples in the MPE measurement, significant Joule heating should take place and may break the samples; this is unlikely to occur for insulating samples where the traditional piezoelectric effect is observed. The very high efficiency of the MPE in EuMnBi_2_ as a piezoelectric response was missed in our previous paper^9^, since the amplitude of the electric current flowing inside the sample was not correctly estimated; the set voltage magnitude of a function generator (constant voltage source) was used for the estimation of the effective piezoelectric coefficient. Note that since the magnitude of the generated strain is very small, the influence of the MPE on the physical property of the material should be negligible during the MPE measurement.

Here, we need to note that the effective piezoelectric coefficient may be overestimated due to an experimental artifact in the present MPE measurement. The magnitude of the MPE displacement signal depends slightly on laser positions and sample-surface conditions as discussed for Fig. 2c, and also it can be subject to stiffness of the bonding material between the sample and the Cu sample holder. We have used GE varnish to avoid Joule heating effects during the MPE measurements^10^, and the deformation of the backside face of the EuMnBi_2_ sample may affect the detected magnitudes of the MPE signal.

Another possible artifact in the estimation of the $\tilde{d}$ value is a contribution from the conventional piezoelectric effect. The conventional piezoelectric effect might also arise in addition to the MPE in EuMnBi_2_, since the electric field inside the sample is non-zero. However, the magnitude of the piezoelectric signal should decrease with decreasing temperature owing to more significant screening in higher conductivity states; this is not the case in Fig. 4. In the present experiment, the conventional piezoelectric effect is unlikely to be observed in the MPE measurement. Furthermore, mechanical resonance has a chance to enhance displacement signals, as reported in micron-scale cantilever samples^25,26^. However, our sample has a millimeter thickness, and resonance frequency is not recognized around 10 kHz in the frequency spectra at 0 mA (Fig. 3b). Hence, though we need to be still careful about the precise estimation of the MPE efficiency, the MPE has a high potential as an alternative to piezoelectricity. Compared to traditional piezoelectric effects, low-temperature application could be suitable, since higher conductivity leads to a higher efficiency of the MPE. The improvement of the stability of the measurement system will also enable the determination of all the components of the MPE coefficient tensor. Further study of the MPE should be important as a new research direction for exploring new piezoelectric materials.

## Methods

### Material growth

Single crystals of EuMnBi_2_ were grown by a Bi self-flux method following the previous paper^12^. High-purity ingots of Eu (99.9%), Mn (99.9%), and Bi (99.999%) were mixed in the ratio of Eu/Mn/Bi = 1:1:9 and put into an alumina crucible in an argon-filled glove box. The crucible was sealed in an evacuated quartz tube and heated at 1000 °C for 10 hours, followed by slow cooling to 400 °C at the rate of ~2 °C /h, where the excess Bi flux was decanted with use of a centrifuge. Millimeter-size plate-like single crystals were obtained. The largest plane of as-grown crystals faces the crystallographic (001) plane. We checked the crystallographic directions of the sample with a Laue diffraction technique, and cut the sample with a wire saw, so that (110) planes were obtained as shown in Fig. 2b. The length along the [001] direction is 0.9 mm, and that along the [110] direction is 1.4 mm.

### Measurement of magnetization and resistivity

Magnetization and resistivity were measured down to 1.9 K using a Magnetic Property Measurement System (Quantum Design, Inc.) and a Physical Properties Measurement System (Quantum Design, Inc.), respectively. Temperature dependence of magnetization was measured under the magnetic field of 0.1 T applied along the [001] direction. The magnetization measurement was performed in the field-cooling condition. Temperature dependence of in-plane resistivity *ρ*_*xx*_ was measured without magnetic fields by a conventional four-terminal method. In making electrodes, special care was taken to avoid the admixture of the interlayer resistance^12^. Temperature dependence of interlayer resistivity *ρ*_*zz*_ was also measured in zero field by a four-terminal method on bar-shaped samples. Current terminals were formed so as to completely short out the in-plane current^12^. The reproducibility of the resistivity data was confirmed for several samples.

### Measurement of the magneto-piezoelectric effect

In the measurement of the magneto-piezoelectric effect, AC electric currents were applied to the sample, and AC displacements generating in response to the AC electric current were measured using a laser Doppler vibrometer (Onosokki CO., LTD). The EuMnBi_2_ sample was fixed on a Cu holder of a liquid-He optistat (Cryo Industries of America, Inc.) with GE varnish. To apply electric currents, electrodes were formed on the (001) planes of the EuMnBi_2_ sample with conductive Ag paste. The size of the electrodes is approximately 2.5 mm^2^. A constant current source (model k6221, Tektronics, Inc.) was used to apply AC electric currents. The liquid-He optistat has a quartz window through which the laser was irradiated from the laser Doppler vibrometer. The Doppler frequency shift of the laser reflected from the (110) top surface of the sample was detected using a two-beam laser interferometer inside the laser Doppler vibrometer, and the velocity of the sample vibration was extracted. The velocity data was transferred to an FFT analyzer (Onosokki CO., LTD) and converted to displacement data by numerically integrating the velocity with respect to time. The lowest temperature of the measurement was 4.5 K, and higher temperatures than 4.5 K were obtained with a heater attached beneath the sample holder in the optistat into which liquid He was continuously flowed.
